# Assessment of area and structural irregularity of retinal layers in diabetic retinopathy using machine learning and image processing techniques

**DOI:** 10.1038/s41598-024-54535-6

**Published:** 2024-02-18

**Authors:** Hamid Riazi-Esfahani, Behzad Jafari, Hossein Azimi, Masoud Rahimi, Jamshid Saeidian, Parnia Pouya, Hooshang Faghihi, Arash Mirzaei, Esmaeil Asadi Khameneh, Elias Khalili Pour

**Affiliations:** 1 Retina Ward, Farabi Eye Hospital, Tehran University of Medical Sciences, South Kargar Street, Qazvin Square, Tehran, Iran; 2 Faculty of Mathematical Sciences and Computer, Kharazmi University, No. 50, Taleghani Ave, Tehran, Iran; 3 Research Center for Evidence-Based Medicine, Health Management and Safety Promotion Research Institute, Tabriz University of Medical Sciences, Tabriz, Iran

**Keywords:** Diagnostic markers, Diagnostic markers, Eye diseases

## Abstract

Diabetes retinopathy prevention necessitates early detection, monitoring, and treatment. Non-invasive optical coherence tomography (OCT) shows structural changes in the retinal layer. OCT image evaluation necessitates retinal layer segmentation. The ability of our automated retinal layer segmentation to distinguish between normal, non-proliferative (NPDR), and proliferative diabetic retinopathy (PDR) was investigated in this study using quantifiable biomarkers such as retina layer smoothness index (SI) and area (S) in horizontal and vertical OCT images for each zone (fovea, superior, inferior, nasal, and temporal). This research includes 84 eyes from 57 individuals. The study shows a significant difference in the Area (S) of inner nuclear layer (INL) and outer nuclear layer (ONL) in the horizontal foveal zone across the three groups (p < 0.001). In the horizontal scan, there is a significant difference in the smoothness index (SI) of the inner plexiform layer (IPL) and the upper border of the outer plexiform layer (OPL) among three groups (p < 0.05). There is also a significant difference in the area (S) of the OPL in the foveal zone among the three groups (p = 0.003). The area (S) of the INL in the foveal region of horizontal slabs performed best for distinguishing diabetic patients (NPDR and PDR) from normal individuals, with an accuracy of 87.6%. The smoothness index (SI) of IPL in the nasal zone of horizontal foveal slabs was the most accurate at 97.2% in distinguishing PDR from NPDR. The smoothness index of the top border of the OPL in the nasal zone of horizontal slabs was 84.1% accurate in distinguishing NPDR from PDR. Smoothness index of IPL in the temporal zone of horizontal slabs was 89.8% accurate in identifying NPDR from PDR patients. In conclusion, optical coherence tomography can assess the smoothness index and irregularity of the inner and outer plexiform layers, particularly in the nasal and temporal regions of horizontal foveal slabs, to distinguish non-proliferative from proliferative diabetic retinopathy. The evolution of diabetic retinopathy throughout severity levels and its effects on retinal layer irregularity need more study.

## Introduction

Diabetic retinopathy is the primary cause of vision loss among adults of working age. The increasing diabetic population will contribute to a significant public health concern regarding visually impaired individuals^1^. Diabetes is associated with changes in the retinal microvasculature and structure, which can gradually result in visual impairment. The prevention of vision loss caused by diabetic retinopathy necessitates prompt diagnosis, frequent monitoring, and timely therapeutic intervention^2,3^. One major obstacle is the identification of diabetic individuals who are at risk of developing retinopathy and advancing to vision-threatening conditions such as macular edema or proliferative diabetic retinopathy^3–5^.

Diabetic retinopathy can be categorized into two primary classes: nonproliferative and proliferative. The term “proliferative” pertains to the presence or absence of neovascularization, which denotes abnormal growth of blood vessels in the retina^6^. Non-proliferative diabetic retinopathy NPDR refers to the initial stage of disease in which neovascularization is absent. As the disease advances, it can develop into proliferative diabetic retinopathy PDR, characterized by neovascularization and a higher risk of significant visual complications^7,8^.

The progression of diabetic retinopathy can be averted through effective metabolic control, timely detection, and early treatment of DR. Fluorescein angiography (FA) is the preferred imaging technique for identifying retinal neovascularization and differentiating between NPDR and PDR^9,10^. Fluorescein angiography (FA) is an invasive imaging technique that necessitates the administration of intravenous dye and is associated with systemic and allergic complications^11^. Optical coherence tomography (OCT) is a non-invasive imaging technique used to visualize structural changes in the retina and choroid. It is commonly employed in the examination of different retinal diseases including diabetic retinopathy, age-related macular degeneration (AMD), retinal vein occlusion, and vitreomacular interface disorders^9^. OCT offers a variety of biomarkers for diagnosing, treating, and monitoring retinal changes in patients. Another advantage of this imaging modality is its ability to provide high-resolution images of the retinal layers structure, distinguishing it from FA^9,12^.

The healthy retina exhibits an organized and layered structure on spectral-domain OCT (SD-OCT), which is characterized by diverse reflectance patterns. With the advancement of SD-OCT knowledge, various imaging biomarkers have been proposed to assess visual prognosis. These include central retinal thickness, which provides insight into the entire retina, as well as the attenuation of the ellipsoid zone or external limiting membrane, which indicates photoreceptors dysfunction^13,14^. Additionally, the disorganization of the retinal inner layers (DRIL) is defined as the inability to distinguish between the ganglion cell layer–inner plexiform layer complex, inner nuclear layer, and outer plexiform layer. The inner retinal layers encompass axons, bipolar cells, and amacrine cell nuclei, all crucial for transmitting visual signals from photoreceptors to the ganglion cell layer. The DRIL indicates impairment in these components, resulting in abnormal visual signal processing^15^. Similarly, the presence and localization of hyperreflective foci (HF) within the retina may have a similar prognostic value^16^. The utilization of new OCT biomarkers could enhance risk stratification, aid in characterizing disease morphology, optimize the use of specific therapies such as anti-vascular endothelial growth factor (VEGF) therapy, improve prognosis counseling, and provide more precise inclusion criteria for therapeutic trials^17,18^.

Segmentation of retinal layers is necessary for evaluating OCT images of the retinal structure. There are various methods to accomplish this task, including manual, semi-automated, or fully automated approaches^19–23^. In a recent study, an automated technique was developed to serve as a dependable, expeditious, and user-friendly method for segmenting the inner plexiform layer (IPL) and outer plexiform layer (OPL), as well as estimating the smoothness index (SI) inside these specific layers^17^. Automated segmentation methods are advantageous due to their ability to operate continuously and independently of user experience, thereby mitigating the time-consuming and low repeatability nature of manual segmentation procedures^17,19,23^.

The IPL, or inner synaptic layer, comprises synaptic connections formed by the axons of bipolar cells and the dendrites of ganglion cells. The OPL, or outer synaptic layer, consists of synapses connecting photoreceptor cells with cells from the inner nuclear layer. The external limiting membrane (ELM) forms the junctional complex between Müller glia and photoreceptor cells^24^. We postulated that synaptic or junctional levels might be the initial sites of manifestation for abnormalities in the irregularity of retinal layers. The purpose of this study was to determine how well our previously developed automated retinal layer segmentation performed in differentiating between normal, NPDR, and PDR using quantified biomarkers like irregularity of hyperreflective retinal layers through machine learning techniques.

## Methods

The study was conducted at Farabi Eye Hospital, Tehran University of Medical Sciences, Iran, and followed the Declaration of Helsinki’s principles. The institutional review board of Tehran University of Medical Sciences approved the study (IR.TUMS.FARABIH.REC.1400.028). The patients all provided written informed consent.

Both normal subjects without diabetes mellitus and patients with diabetic retinopathy were recruited. All patients with DR underwent fluorescein angiography (Heidelberg Engineering, Heidelberg, Germany) and SD-OCT (RTVue XR 100 Avanti device manufactured by Optovue, Inc. in Fremont, CA, USA) at baseline. Patients diagnosed with diabetic retinopathy were categorized into two groups: NPDR and PDR. This categorization was determined by the presence or absence of neovascularization in the retina or optic disc, as observed during a dilated fundus examination, as well as the presence of dye leakage on fluorescein angiography (FA). Therefore, the retinopathy stage was consensually determined by two experienced retina specialists (H. R. E. and E. K. P.) after evaluating fundus examination findings and fluorescein angiography images.

Patients with diabetic macular edema (DME) were excluded due to its potential to induce irregularities in the retinal layer structure, which could introduce bias into the findings of this study. Other exclusion criteria encompassed the existence of exudate and fibrovascular proliferation in the macular region, as well as other macular diseases such as age-related macular degeneration, macular dystrophies, and vitreomacular interface disorders. Additionally, uveitis, uncontrolled glaucoma, severe media opacity, visual acuity below 20/200, and refractive error exceeding + 3 or less than − 3 were also considered as exclusion criteria. Participants with a documented medical background involving prior PRP (panretinal photocoagulation), macular photocoagulation, intravitreal anti-VEGF (vascular endothelial growth factor) injections, or intraocular surgery, with the exception of cataract extraction, were not included in the study. The study excluded pictures of low quality, defined as having a signal strength index (SSI) below 40 as shown by the quality evaluation report generated by the RTVue program. The images were obtained using both horizontal and vertical raster patterns over the foveal region measuring 8 mm by 12 mm. All images had dimensions of 256 × 728 pixels.

The retinal layers were segmented automatically using the method described in our recently published study^17^. The automated procedure entailed mitigating artifacts and enhancing image quality through the utilization of nonlocal algorithms, notably Gaussian filters with a kernel size of 11 × 11. Subsequently, Gabor filters with two distinct kernel sizes of 6 × 6 were employed to specifically focus on the retinal area. The details were previously described in our published paper, where we supplied the Gabor filter formula and associated parameters in a companion table^17^. In all horizontal OCT scans, an unwanted region known as the optic disc is evident. We automatically trimmed images in all horizontal scans to eliminate the optic disc in SI segmentation and quantification. The boundaries of the retinal layers were then recognized using Support Vector Regression (SVR), a well-known machine learning approach. SVR is a supervised learning technique that employs points on layers and a prediction function. In the event of mislabeling, the model predicts and substitutes the wrong values, which is a big benefit of utilizing SVR.

The data was translated into a higher-dimensional space known as a kernel because our model used a nonlinear function. In SVR implementation, many kernels such as polynomials, sigmoid, Radial Basis Functions (RBF), and wavelet functions are used. The kernel used has a significant impact on the efficacy of SVR. Notably, the kernel-based method can use feature mapping with an infinite number of dimensions. The wavelet kernel is supposed to generate more appropriate outcomes heuristically. As a result, the algorithm extracted the retina’s hyperreflective layers (IPL, OPL, and EZ).

**Figure 1:**
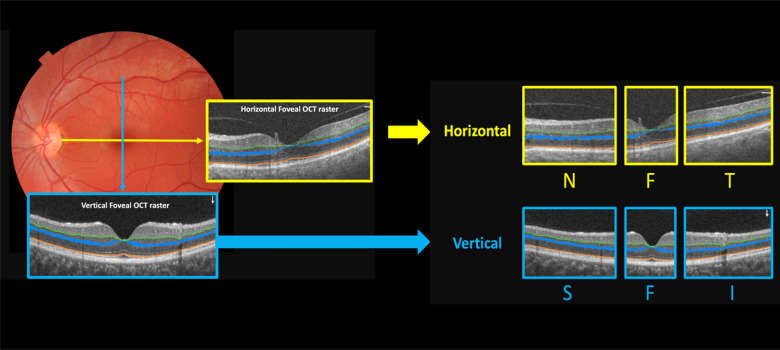
The provided illustration depicts the cross-sectional sections of the fovea and their respective subdivisions. The blue arrow indicates a vertical slab, while the yellow arrow indicates a horizontal slab. Both the horizontal and vertical slabs are subdivided into three zones. The horizontal cuts are labeled as N for nasal, F for fovea, and T for temporal. Similarly, the vertical cuts are labeled as S for superior, F for fovea, and I for inferior.

The IPL and EZ were depicted as a thin line, while the OPL was depicted as a thick band with an upper and border. The horizontal cross-section OCT images were segmented and extracted into three zones: nasal, foveal (comprises 20% of the central pixels equally located on both the left and right sides of the fovea) and temporal. The vertical cross-sectional OCT images were divided into superior, fovea (comprises 20% of the central pixels equally located on both the upper and lower sides of the fovea), and inferior zones in vertical diameter. Figure 1 displays the divisions and patterns of the cross-sections.

In the next stage, the biomarkers, specifically the smoothness index (SI), and area (S) of retina layers were calculated individually for each zone (fovea, superior, inferior, nasal, and temporal).

**Figure 2:**
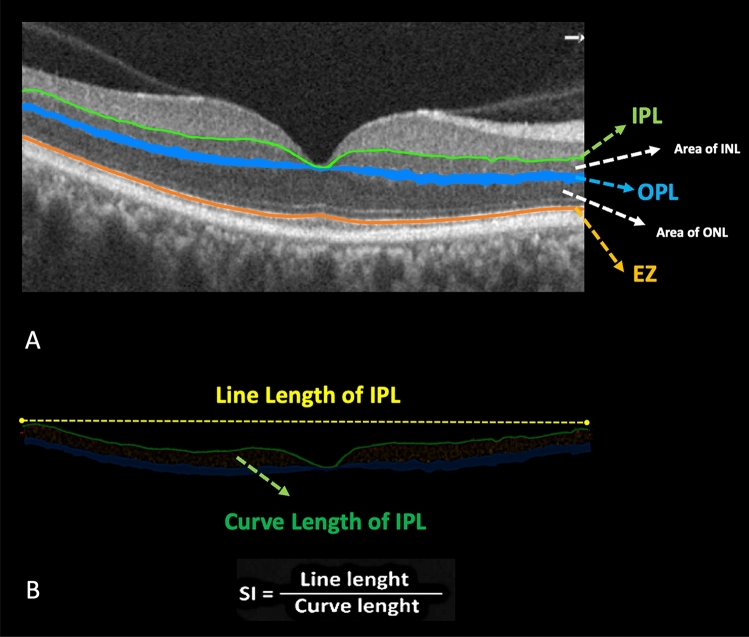
(**A**) illustrates the zones and areas that have been assessed in the present investigation. The white dashed arrows depict areas (S) of the inner nuclear layer (INL) and outer nuclear layer (ONL). The inner plexiform layer (IPL) is depicted as a linear representation by the green arrow. The Blue band represents the outer plexiform layer (OPL). The upper and lower boundaries of the outer plexiform layer (OPL) have been assessed individually, and the measurement of the distance between these boundaries has been designated as the OPL area. Moreover, the orange line represents the ellipsoid zone (EZ). (**B**) In this section, we have presented the calculation of the smoothness index (SI). The line length, shown by the yellow dashed line, represents the Euclidean distance between the starting and ending points of the IPL. On the other hand, the curve length, depicted by the green curve, represents the actual length of the IPL.

The SI of each layer was determined by dividing the line length (LL) of that retinal layer, which represents the direct distance between the start and end points of the layer in a specific zone, by the curve length (CL), which represents the actual length of the segmented layer in that zone. The SI was calculated for IPL, OPL, and EZ extracted bands. The OPL measurements involved calculations of the SI for the upper borders. The formula for calculating the SI is depicted in Fig. 2.

$$\mathrm{SI }= \frac{Line\, Length (LL)}{Curve\, Length (CL)}.$$

The area of the inner nuclear layer (INL) can be determined by calculating the area between the inner plexiform layer (IPL) and the upper boundary of the outer plexiform layer (OPL). The area of the outer plexiform layer (OPL) was determined by calculating the area between the upper and lower borders of the OPL. The area of the outer nuclear layer (ONL) was determined by calculating the region between the lower boundary of the outer plexiform layer (OPL) and the ellipsoid zone (EZ). Figure 2 illustrates the IPL, OPL, and EZ extracted layers as well as the INL, OPL and ONL area.

Diabetic retinopathy was classified as either normal, NPDR, or PDR based on the severity of the disease. Each person’s SI and area (S) of the segmented layers were automatically measured in the OCTs’ aforementioned zones, and the results were compared across groups.

### Ethical approval

This study has been approved by the local institutional review board of Tehran University of Medical Sciences (IR.TUMS.FARABIH.REC.1400.028).The study was performed in accordance with the Helsinki Declaration of 1964, and its later amendments.

### Informed consent

All patients provided informed consent to participate in the study.

## Statistical methods

To present the data, we used mean, standard deviation (SD), median and range, frequency, and percentage. To compare variables between groups considering the correlation of measurements in two eyes, we used a generalized estimating equation (GEE). All the analysis was adjusted based on age and sex. To assess the diagnostic ability of the variables we used the ROC curve and the associated 95% CI. The Spearman correlation coefficient was applied to evaluate the association of the quantitative variables. Statistical analysis was performed with SPSS (IBM Corp. Released 2020. IBM SPSS Statistics for Windows, Version 27.0. Armonk, NY: IBM Corp). A p-value less than 0.05 is considered statistically significant.

## Results

In the present investigation, a total of 84 eyes from 57 patients were included. Table 1 presents the demographic distribution of various groups of patients participating in the present investigation. The participants’ average age was 52 ± 11.2 years, with 36 eyes (42.8%) belonging to males.

**Table 1:** Presents the demographic data among various groups of patients participating in the present investigation.

	Normal (n = 33)	NPDR (n = 36)	PDR (n = 15)	Total (n = 84)	p-value
Men, n (%)	17 (51.5)	14 (38.8)	5 (33.3)	36 (42.8)	0.12^a^
Right eye, n (%)	15 (45.4)	19 (52.7)	9 (60)	43 (51.1)	0.76^a^
Age (years), mean ± SD	45 ± 11.3	56 ± 13.1	57 ± 9.6	52 ± 11.2	0.42^b^
log MAR BCVA, mean ± SD	0.12 ± 0.12	0.36 ± 0.42	0.45 ± 0.47	0.31 ± 0.33	0.09^b^

*BCVA* best-corrected visual acuity, *NPDR* non-proliferative diabetic retinopathy, *PDR* proliferative diabetic retinopathy.

^a^Chi-square tests.

^b^Independent t-test.

The first group included 33 normal optical coherence tomography (OCT) scans obtained from a sample of 33 individuals, with 51.5% of the subjects identifying as male. The second group included 36 optical coherence tomography (OCT) images obtained from a group of 36 individuals diagnosed with NPDR, of whom 38.8% were male. The final cohort included a total of 15 optical coherence tomography (OCT) images obtained from 15 individuals, of whom 33.3% were male, all diagnosed with PDR.

Tables 2 and 3 present the average and standard deviation of measurements for each variable across three distinct groups (normal, NPD, and PDR). Additionally, these tables include pairwise comparisons among the three groups.

**Table 2:** In horizontal optical coherence tomography slabs, this table presents the average and standard deviation of observations for each variable across three separate groups (normal, NPDR, and PDR).

		Group			p-value	Multiple comparisions		
		Normal	NPDR	PDR		P1	P2	P3
Horizontal slabs—foveal zone	S_INL.H.fovea	3357.44 ± 866.07	5179.56 ± 1169.94	5316.13 ± 1598.35	** < 0.001**	** < 0.001**	** < 0.001**	0.91578
	S_ONL.H.fovea	24,879.87 ± 3237.89	29,462.08 ± 5319.02	29,361.67 ± 2845.06	** < 0.001**	** < 0.001**	** < 0.001**	0.99998
	S_OPL.H.fovea	2307.19 ± 724.41	2604.81 ± 906.79	2599.07 ± 1132.69	0.36402	0.4238	0.8186439	0.99886
	SI_IPL.H.fovea	0.91 ± 0.02	0.91 ± 0.02	0.91 ± 0.02	0.63898	0.96141	0.9112877	0.71803
	SI_OPL_up.H.fovea	0.93 ± 0.02	0.94 ± 0.02	0.93 ± 0.01	0.45537	0.71204	0.999999	0.62501
	SI_EZ.H.fovea	0.95 ± 0.01	0.95 ± 0.01	0.94 ± 0.03	0.2394	0.54337	0.7918036	0.3984
Horizontal slabs—temporal zone	S_INL.H.Temporal	20,271.5 ± 3098.11	21,607.47 ± 3732.75	21,666.21 ± 3334.99	0.61386	0.69012	0.9465641	0.98409
	S_ONL.H.Temporal	54,562.81 ± 6418.32	53,070.61 ± 8910.94	57,912.64 ± 8575.12	0.31518	0.796	0.3751907	0.96437
	S_OPL.H.Temporal	12,490.53 ± 1566	11,237.22 ± 1747.79	11,354.5 ± 3125.23	0.06509	0.05956	0.6756801	0.95759
	SI_IPL.H.Temporal	0.93 ± 0.01	0.93 ± 0.01	0.9 ± 0.02	** < 0.001**	0.76157	** < 0.001**	** < 0.001**
	SI_OPL_up.H.Temporal	0.93 ± 0.01	0.93 ± 0.01	0.91 ± 0.02	**0.00196**	0.48475	**0.0014818**	**0.01183**
	SI_EZ.H.Temporal	0.94 ± 0.02	0.94 ± 0.01	0.93 ± 0.02	0.37693	0.71234	0.9406616	0.51626
Horizontal slabs—nasal zone	S_INL.H.Nasal	22,617.88 ± 3607.72	23,088.25 ± 3417.13	23,575.07 ± 3849.62	0.21948	0.7038	0.2577146	0.77395
	S_ONL.H.Nasal	58,425.69 ± 7715.16	60,214.31 ± 12,619.52	60,470.47 ± 7431.86	0.32646	0.65075	0.8607225	0.40356
	S_OPL.H.Nasal	13,122.72 ± 2189.31	12,177.72 ± 2775.48	12,287.4 ± 2189.84	0.05676	0.13685	0.1066176	0.96035
	SI_IPL.H.Nasal	0.93 ± 0.01	0.93 ± 0.01	0.89 ± 0.03	** < 0.001**	0.72501	** < 0.001**	** < 0.001**
	SI_OPL_up.H.Nasal	0.93 ± 0.01	0.93 ± 0.01	0.9 ± 0.03	**0.00392**	0.99918	**0.0037228**	**0.00281**
	SI_EZ.H.Nasal	0.94 ± 0.01	0.94 ± 0.01	0.94 ± 0.02	0.81829	0.98439	0.8987655	0.94468

Also provides pairwise comparisons between the three groups. We utilized a generalized estimating equation (GEE) to compare variables between groups while accounting for the correlation of data in two eyes.

*S* surface area, *SI* smoothness index, *INL* inner nuclear layer, *ONL* outer nuclear layer, *IPL* inner plexiform layer, *OPL* outer plexiform layer, *EZ* ellipsoid zone, *up* upper boundary of IPL.

Statistically significant outcomes are shown in bold font. P1: normal vs NPDR, P2: normal vs PDR, P3: NPDR vs PDR.

**Table 3:** In vertical optical coherence tomography slabs, this table presents the average and standard deviation of observations for each variable across three separate groups (normal, NPDR, and PDR).

		Group			p-value	Multiple comparisions		
		Normal	NPDR	PDR		P1	P2	P3
Vertical slabs—foveal zone	S_INL.V.fovea	6733 ± 1753	5856 ± 1402	7022 ± 2247	**0.038**	0.138	0.989	0.111
	S_onl.V.fovea	29,166 ± 3910	27,450 ± 4772	26,275 ± 3956	**0.026**	0.269	**0.032**	0.75
	S_OPL.V.fovea	4453 ± 1009	4193 ± 1228	5711 ± 1575	**0.003**	0.888	**0.015**	**0.002**
	SI_IPL.V.fovea	0.918 ± 0.026	0.912 ± 0.024	0.914 ± 0.031	0.566	0.636	0.934	0.982
	SI_OPL_up.V.fovea	0.942 ± 0.016	0.936 ± 0.015	0.924 ± 0.034	0.066	0.253	0.125	0.476
	SI_EZ.V.fovea	0.946 ± 0.011	0.949 ± 0.013	0.937 ± 0.034	0.233	0.742	0.503	0.309
Vertical slabs—inferior zone	S_INL.V.INF	22,476 ± 3243	23,461 ± 3611	24,563 ± 4926	0.261	0.589	0.364	0.825
	S_onl.V.INF	50,752 ± 6300	47,902 ± 10,501	53,080 ± 11,555	0.167	0.37	0.71	0.229
	S_OPL.V.INF	13,038 ± 2148	14,512 ± 2950	16,054 ± 2926	**0.001**	0.074	**0.001**	0.23
	SI_IPL.V.INF	0.927 ± 0.015	0.928 ± 0.013	0.932 ± 0.013	0.584	0.998	0.698	0.764
	SI_OPL_up.V.INF	0.932 ± 0.013	0.93 ± 0.015	0.926 ± 0.017	0.418	0.813	0.525	0.919
	SI_EZ.V.INF	0.942 ± 0.018	0.938 ± 0.015	0.942 ± 0.014	0.722	0.843	0.998	0.911
Vertical slabs—superior zone	S_INL.V.SUP	22,792 ± 3087	22,941 ± 4454	23,060 ± 3145	0.973	1	0.994	0.998
	S_onl.V.SUP	52,050 ± 6720	51,708 ± 8842	54,366 ± 10,127	0.661	0.998	0.777	0.755
	S_OPL.V.SUP	14,599 ± 2191	15,487 ± 3507	16,400 ± 3161	0.12	0.486	0.166	0.778
	SI_IPL.V.SUP	0.936 ± 0.015	0.931 ± 0.015	0.929 ± 0.02	0.284	0.488	0.401	0.955
	SI_OPL_up.V.SUP	0.934 ± 0.015	0.928 ± 0.029	0.929 ± 0.02	0.378	0.552	0.626	0.993
	SI_EZ.V.SUP	0.946 ± 0.013	0.946 ± 0.014	0.942 ± 0.021	0.8	1	0.885	0.898

Also provides pairwise comparisons between the three groups. We utilized a generalized estimating equation (GEE) to compare variables between groups while accounting for the correlation of data in two eyes.

*S* surface area, *SI* smoothness index, *INL* inner nuclear layer, *ONL* outer nuclear layer, *IPL* inner plexiform layer, *OPL* outer plexiform layer, *EZ* ellipsoid zone, *up* upper boundary of IPL.

Statistically significant outcomes are shown in bold font. P1: normal vs NPDR, P2: normal vs PDR, P3: NPDR vs PDR.

The analysis of the tables reveals a statistically significant difference in the area (S) of the INL and ONL in the horizontal foveal zone among the three groups (p < 0.001). However, upon conducting pairwise comparisons, this difference is observed only between the normal group and the NPDR group (p < 0.001), as well as between the normal group and the PDR group (p < 0.001). No significant difference is observed between the NPDR and PDR groups.

Regarding variables in the temporal zone, there is a statistically significant difference in the smoothness index (SI) of the IPL and the upper border of the OPL among three groups in the horizontal scan (p < 0.001 and p = 0.0019 for IPL and OPL, respectively). However, upon pairwise comparison, this difference is only observed between the normal and PDR group (p < 0.05), as well as between the NPDR and the PDR group (p < 0.05), but not between the normal and NPDR groups.

Regarding the variables in the horizontal nasal zone, A significant statistical difference can be observed in the smoothness index (SI) of the IPL and the upper border of the OPL among the three groups (p < 0.05). However, upon conducting pairwise comparisons, this difference is only observed between the normal and PDR group (p < 0.05), as well as between the NPDR group and the PDR group (p < 0.05), but not between the normal and NPDR groups.

Regarding the variables pertaining to the vertical slabs, the area (S) of the OPL in the foveal zone exhibited a statistically significant difference among the three groups (p = 0.003). However, upon conducting pairwise comparisons, this difference was only observed between the normal and PDR group (p = 0.015), as well as between the NPDR and the PDR group (p = 0.002), but not between the normal and NPDR groups.

The remaining variables, as indicated in Tables 2 and 3, did not exhibit statistically significant changes or demonstrated significant changes only between two specific groups in the pairwise comparison.

In order to evaluate the diagnostic efficacy of the variables, we employed the receiver operating characteristic (ROC) curve and calculated the corresponding 95% confidence interval (CI).

**Figure 3:**
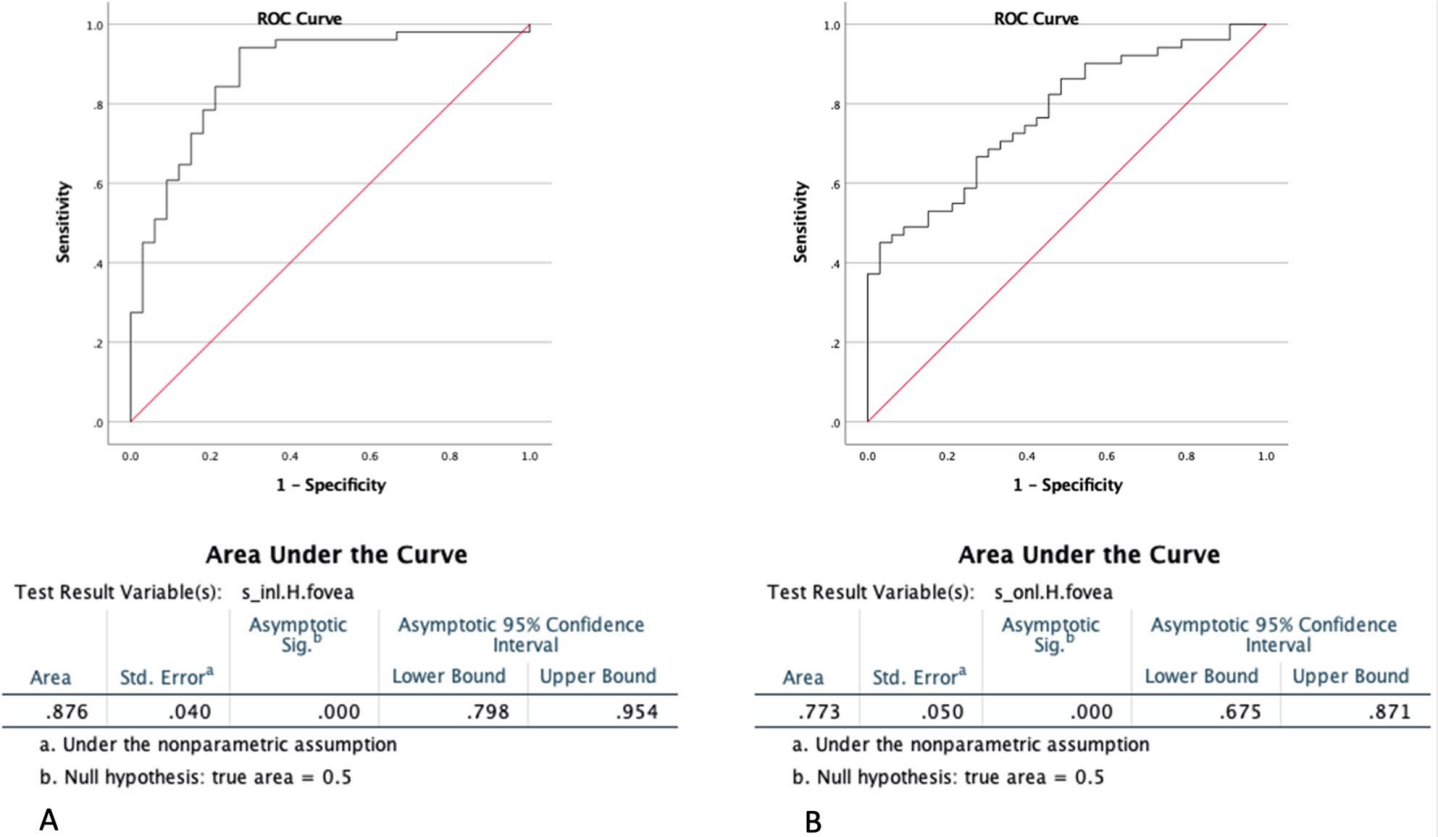
The receiver operating characteristic curves (ROC curves). (**A**) To differentiate between diabetic patients (NPDR and PDR) and normal patients, the study found that the area (S) of the inner nuclear layer (INL) in the foveal zone of horizontal slabs exhibited the most effective performance. This measure achieved an accuracy rate of 87.6% (confidence interval 0.798–0.954). (**B**) The accuracy of discriminating between individuals with normal health conditions and those diagnosed with Diabetes, based on the area of the outer nuclear layer (ONL) in the foveal zone of horizontal slabs, was found to be 77.3% (confidence interval 0.675–0.871).

For discriminating diabetic patients (NPDR and PDR) from normal patients, the area (S) of the inner nuclear layer (INL) in the foveal region of horizontal slabs demonstrated the highest performance, achieving an accuracy of 87.6% (CI 0.798–0.954) (Fig. 3A). The area of ONL in the foveal region of horizontal slabs demonstrated an accuracy of 77.3% (confidence interval: 0.675–0.871) in distinguishing between those with normal health conditions and those diagnosed with Diabetes (Fig. 3B).

**Figure 4:**
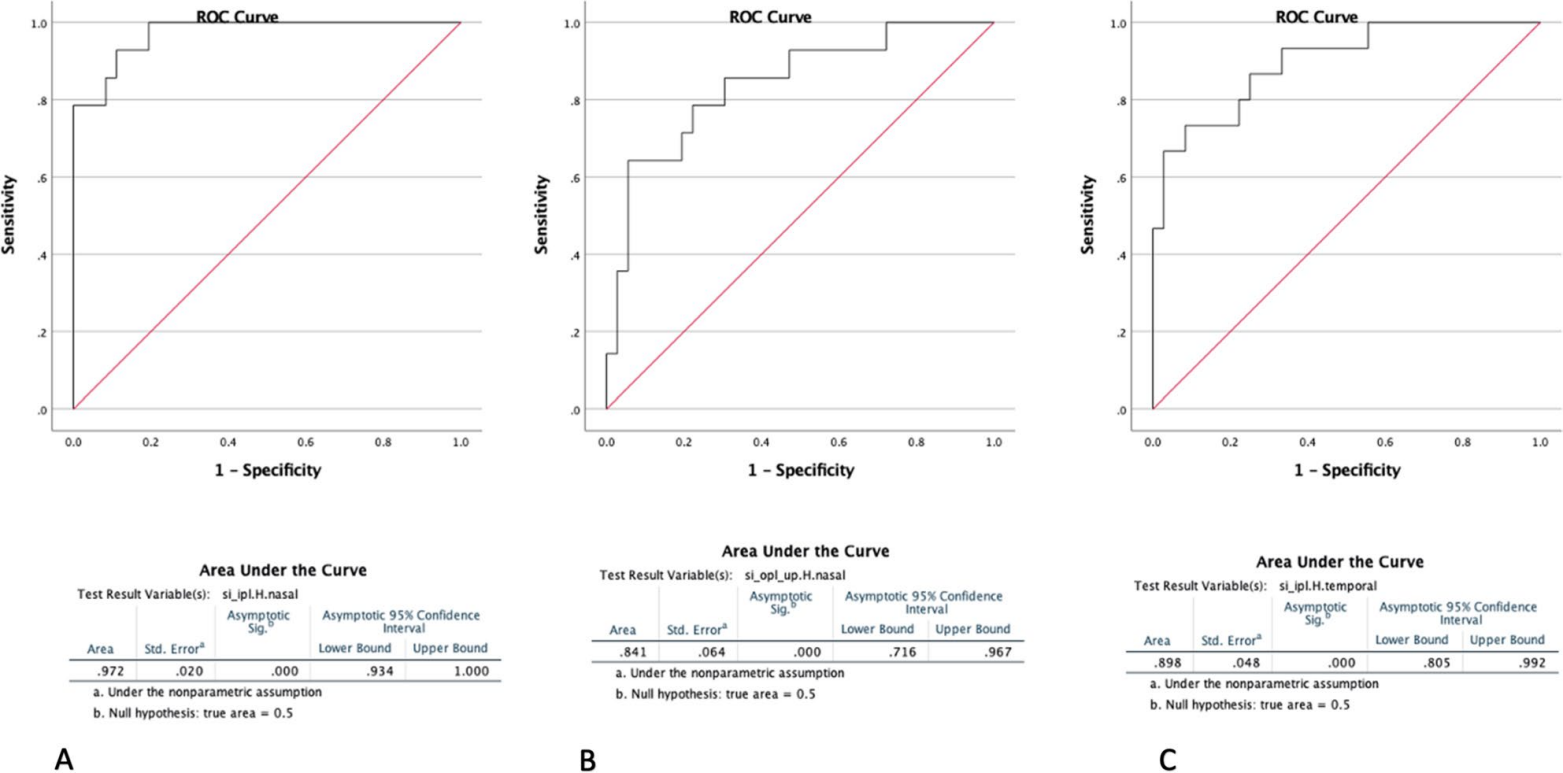
The receiver operating characteristic curves (ROC curves). (**A**) In the context of discriminating between patients with PDR and NPDR, the smoothness index (SI) of IPL in the nasal zone of horizontal foveal slabs achieved the highest level of performance, with an accuracy of 97.2% (confidence interval 0.934–1.00). (**B**) Demonstrates that the accuracy of distinguishing between patients with NPDR and patients with PDR based on the smoothness index of the upper border of the OPL in the nasal zone of horizontal slabs was 84.1% (CI 0.716–0.967). (**C**) Demonstrates that the smoothness index of IPL in the temporal zone of horizontal slabs distinguished between patients with NPDR and those with PDR with an accuracy of 89.8% (CI 0.805–0.992).

In the context of discriminating between patients with PDR and NPDR, the smoothness index (SI) of IPL in the nasal zone of horizontal foveal slabs demonstrated the highest level of performance, achieving an accuracy of 97.2% (confidence interval 0.934–1.00) (Figure-4A). The accuracy of discriminating between patients with NPDR and patients with PDR based on the smoothness index of the upper border of the OPL in the nasal zone of horizontal slabs was found to be 84.1% (CI 0.716–0.967) (Fig. 4B). The study found that the smoothness index of IPL in the temporal zone of horizontal slabs had an accuracy of 89.8% (CI 0.805–0.992) in distinguishing between patients with NPDR and those with PDR (Fig. 4C).

The vertical receiver operating characteristic (ROC) curves, used to distinguish between normal and diabetic individuals, as well as between patients with PDR and NPDR, did not exhibit an area under the curve (AUC) over 80% for any of the variables.

**Figure 5:**
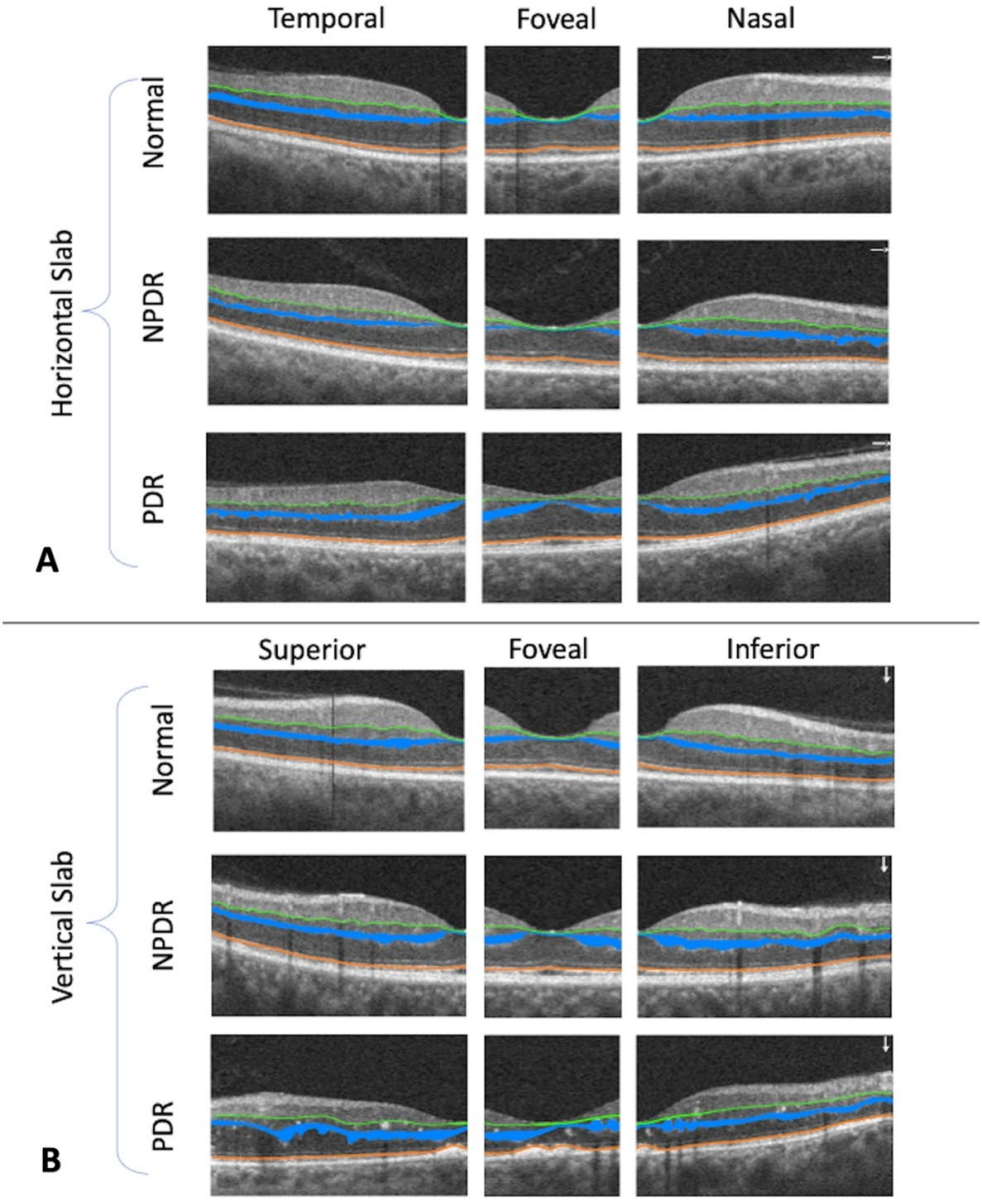
Illustrates the segmentation of IPL (shown by a green line), OPL (represented by a blue region), and ELM (shown by an orange line) in three groups of patients (normal, NPDR, and PDR) in both horizontal (**A**) and vertical (**B**) OCT slabs.

Figure 5 depicts the segmentation of IPL, OPL, and ELM in three groups of patients (normal, NPDR, and PDR) who participated in the current study.

## Discussion

Diabetic retinopathy is a microvascular condition that arises from diabetes mellitus, exhibiting distinctive alterations in both the structural and functional aspects of the retinal layers^25–27^. Optical Coherence Tomography (OCT) has emerged as a highly important modality for non-invasive imaging of retinal layers, offering exceptional resolution and enabling meticulous examination of structural modifications. Emerging optical coherence tomography (OCT) technologies, namely spectral domain OCT (SD-OCT) and swept-source OCT (SS-OCT), offer improved visibility of retinal layers, hence facilitating a more precise assessment of irregularity and area of retinal layers^28,29^. Nevertheless, it is imperative to tackle obstacles such as segmentation errors and discrepancies across optical coherence tomography (OCT) instruments in order to provide uniform measurements across diverse clinical environments^17,19,21^.

In the present investigation, employing a pre-existing automated system for the segmentation of hyperreflective retinal layers, we assessed the irregularity and surface area of the inner retinal layers (such as the inner plexiform layer and inner nuclear layer) as well as the outer retinal layers (such as the outer plexiform layer and outer nuclear layer).

The study investigated differences in smoothness index (SI) between three groups in the IPL and upper border of the OPL, both in temporal and nasal regions. Statistical significance was found, with significant differences between normal and PDR groups, as well as between NPDR and PDR groups, but not between normal and NPDR groups. Notably, the SI of IPL in the nasal area of horizontal foveal slabs achieved 97.2% accuracy in distinguishing PDR from NPDR, while the SI of the upper border of the OPL in nasal horizontal slabs was 84.1% accurate in differentiating NPDR from PDR. Moreover, the SI of IPL in the temporal area of horizontal slabs accurately distinguished NPDR from PDR with an 89.8% accuracy. The presence of irregularity can be associated with the formation of microaneurysms, impaired blood flow in capillaries, and blood-retinal barrier disruption which cause edema, all of which are indicative of the disease’s severity^30^. The utilization of irregularity measurements as biomarkers in the assessment of diabetic retinopathy can exhibit potential for disease monitoring and therapeutic evaluation^31^. The prompt identification of anomalies and alterations in layer areas might potentially enhance the prompt execution of intervention strategies. Moreover, the use of these markers holds promise in facilitating the prediction of illness progression and the tailoring of personalized treatment approaches. The assessment of the regularity (smoothness) of retinal layers has thus far been conducted through semi-automated or manual methods in the context of ERM. However, the algorithm used in this study presents an opportunity to explore this potential biomarker of retinal layers in different vascular and structural diseases of the retina^32^.

It is noteworthy that our findings indicate that the area (S) of the inner nuclear layer (INL) and outer nuclear layer (ONL) in the foveal region of horizontal slabs exhibit the most superior performance in distinguishing diabetic patients (with [NPDR] and [PDR]) from normal patients. Specifically, the area (S) of the INL achieved an accuracy of 87.6%, while the area (S) of the ONL achieved an accuracy of 77.3%.

The measurement of the surface area of certain layers inside the retina, such as the ganglion cell layer (GCL) and the inner plexiform layer (IPL), can yield significant information on cellular density and structural changes. Prior studies have presented findings that suggest a notable correlation between the reduced surface area in these specific layers and the presence of neuronal degeneration and thinning^33–35^. The investigation of retinal thickness in individuals diagnosed with diabetic retinopathy (DR) in comparison to those without the condition demonstrates considerable heterogeneity in the findings. This variability may be attributed to the dynamic nature of the disease progression and the lack of a consistent pattern or trend. This observation is supported by other studies^36–39^. The reduction in neural tissue leads to a drop in thickness, while there is a possibility of an increase in thickness owing to vascular permeability and inflammation. This potential rise may offset the impact of neurodegeneration on macular thickness, as proposed by Sugimoto et al.^40^. However, it should be noted that an increase in total retinal thickness alone does not provide enough evidence to dismiss the possibility of a related neurodegenerative process. The exclusive reliance on macular thickness as a measure for assessing initial alterations in the retinal structure of individuals with type 2 diabetes mellitus (DM2) is inadequate, since it lacks the necessary sensitivity to identify changes in the microstructure of retinal layers^36,37,41,42^. In light of this rationale, our study aimed to assess the thickness of both horizontal and vertical retinal layers inside foveal slabs in individuals with varying degrees of diabetic retinopathy, while excluding those with any indications of diabetic macular edema, thereby distinguishing our research from prior investigations. Based on the findings of the present study, it appears that the horizontal (not vertical) area of the inner nuclear layer (INL) and outer nuclear layer (ONL) inside the central foveal zone exhibit more precision in distinguishing between those with normal retinal health and those diagnosed with diabetes. In general, the measurement of retinal layers area at various depths holds promise as biomarkers that could be used to track the development and advancement of diabetic retinopathy (DR)^43^.

Our concept for differentiating NPDR and PDR by evaluating anomalies in hyperreflective retinal layers is based on the application of OCT biomarkers such as disorganization of retinal layers (DRIL). On optical coherence tomography (OCT), DRIL is distinguished by the difficulty to clearly determine the boundaries of the inner nuclear layer (INL), outer plexiform layer (OPL), and ganglion cell layer-inner plexiform layer (GCL-IPL) complex. We expected that changes in the smoothness of the retinal layers would occur in patients with severe stages of diabetic retinopathy before the formation of DRIL, as opposed to patients with milder stages of diabetic retinopathy or normal patients without diabetic retinopathy. Because recent research found that DRIL is strongly related with areas of ischemic damage, which corresponds to places where there is no flow in the superficial, middle, and deep capillary plexus, this advancement could be due to variables such as increasing degrees of ischemia^15,16,30,44–48^. To verify this theory, we conducted a cross-sectional study on diabetic retinopathy patients who did not have macular edema. However, until we did a larger, more thoroughly controlled study, we were unable to apply our findings to all diabetic patients.

This study is subject to certain limitations, which is a common characteristic of studies with a small sample size. The initial investigation was carried out on a limited cohort of patients. Consequently, the primary obstacle of differentiating between individuals with severe NPDR and PDR will be tackled in subsequent research of greater magnitude, including a bigger sample size. It is important to note that people with retinal edema or vitreous hemorrhage were excluded from the study due to the limited visual clarity and presence of artifacts in assessing retinal layers. However, it should be acknowledged that these patients do exhibit the underlying pathophysiology of diabetic retinopathy. Consequently, a portion of diabetes patients in routine clinical settings may not get any advantages from the methodology employed in the present study. Due to the extensive distribution of vascular changes resulting from diabetes, it has been shown that over 50% of lesions associated with Diabetic Retinopathy (DR) are situated beyond the boundaries of the seven-standard Early Treatment Diabetic Retinopathy Study (ETDRS) fields. Prior research has indicated that the existence of peripheral retinal lesions may indicate a higher level of severity in diabetic retinopathy (DR) in around 9 to 15% of eyes^49,50^. In the present investigation, the researchers were unable to get ultrawide-field fluorescein angiography (UWF-FA) and instead employed conventional fluorescein angiography in all subjects. Additional research is necessary to directly compare the findings of the present investigation, which relied on more precise angiography tests utilizing ultra-widefield fluorescein angiography (UWF-FA)^51^. Furthermore, it should be noted that the HbA1C levels of the participants included in the study were not adjusted, a factor that has the potential to impact the outcomes of the present investigation.

Our previously published investigation established the segmentation method employed in this experiment, which contains a full discussion of its strengths and limits^17^. The present research focuses on applying the algorithm to determine if a patient is NPDR, PDR, or normal. We compiled relevant literature in a Supplementary Table (Supplementary File 1) and compared datasets and the extent of automation among the offered methods. It is worth mentioning that most previous study used datasets from healthy eyes, but our proposed method was used on a diverse population of patients with PDR, NPDR, and normal eyes. Furthermore, as previously investigated and published in our prior investigation ^17^, the SVR-based segmentation approach is totally automated, providing speed. However, our method, like prior image processing-based segmentation algorithms, may not perform well when layer irregularities and oscillations are significant and layers are indistinguishable, as in DRIL. Another limitation of our study is that the proposed algorithm used in the current study does not segment all retinal layers.

Given that OCT tilt can potentially introduce or eliminate pixels at the start or end of the curve and the straight line in certain patients, we propose that this impact may not be as significant since it affects both lines. However, in order to confirm this hypothesis, it is necessary to evaluate the SI measurement in patients with tilted and flattened OCT sections.

In summary, the evaluation of the smoothness index and irregularity of the inner and outer plexiform layers using optical coherence tomography, particularly in the nasal and temporal regions of horizontal foveal slabs, has the potential to be utilized as a biomarker for distinguishing between non-proliferative and proliferative diabetic retinopathy. Further research is required to examine the inherent progression of diabetic retinopathy across various levels of severity and its impact on the irregularity of distinct retinal layers.

### Supplementary Information

Supplementary Information.

## Data Availability

The datasets generated during and/or analyzed during the current study are available from the corresponding author on reasonable request.
