# Supplementary material for: Assessment of area and structural irregularity of retinal layers in diabetic retinopathy using machine learning and image processing techniques
Source: Sci Rep. 2024 Feb 18;14:4013. doi: 10.1038/s41598-024-54535-6 (PMC10874958; doi:10.1038/s41598-024-54535-6)
Supplement: Supplementary file 1 — Supplementary Information. [file 41598_2024_54535_MOESM1_ESM.docx]

Supplementary file-1: A comprehensive comparison between related works for segmentation of retinal layers.

| N | Author  (Year)  (Country) | Title | Dataset | Method | Number of Layers | Validation Metrics | Automation level |
| --- | --- | --- | --- | --- | --- | --- | --- |
| 1 | Stephanie J. Chiu et al  (2010)  (USA) | Automatic segmentation of seven retinal layers in SDOCT images congruent with expert manual segmentation | Healthy | GT + DP | 8 layers | Layer thickness difference  MAE  GCL-IPL=0.77$\pm$ 0.65 px  OPL=1.48 $\pm$ 1.05 px  (px=3.29 um) | Automatic |
| 2 | Pascal A Dufour et al  (2013)  (Switzerland) | Graph-Based Multi-Surface Segmentation of OCT Data Using Trained Hard and Soft Constraints | Healthy | GT + energy minimization | 6 layers | MAE  IPL-INL=4.67$\pm$ 0.83 um  SE  IPL-INL=-3.59 $\pm$0.93 um  (1px=3.9 um) | Automatic |
| 3 | Zhihong Hu et al  (2013)  (USA) | Multiple layer segmentation and analysis in three-dimensional spectral-domain optical coherence tomography volume scans | Healthy | An automated graph search algorithm | 11 Layers | The overall mean and absolute mean for the 11 surfaces are -0.20 ± 0.53 voxels (-0.76 ± 2.06 μm) and 0.82 ± 0.64 voxels (3.19 ± 2.46 μm), respectively | Automatic |
| 4 | Stephanie J. Chiu et al  (2015)  (USA) | Kernel regression-based segmentation of optical coherence tomography images with diabetic macular edema | Healthy and DME | KR + GT+DP | 8 layers  + fluid | Layer thickness differences  GCL-IPL=4.84$\pm$ 5.12 um  OPL=6.35 $\pm$ 6.11 um  (px= 3.87 um) | Automatic |
| 5 | Jason Kugelman et al  (2018)  (Australia) | Automatic segmentation of OCT retinal boundaries using recurrent neural networks and graph search | Healthy children’s and AMD | RNN-GS | 7 layers of healthy, 3 of AMD | SE  INL/IPL= -0.13 $\pm$ 1.10 px OPL/INL =-0.10 $\pm$ 1.31 px  MAE  INL/IPL =0.56 $\pm$ 0.95 px  OPL/INL = 0.69 $\pm$ 1.12 px  (px= 3.9 μm) | Automatic |
| 6 | Zubin Mishra et al  (2020)  (USA) | Automated Retinal Layer Segmentation Using Graph-based Algorithm Incorporating Deep-learning-derived Information | 45 eyes from 42 patients | Deep Learning – Shortest Path algorithm | 11 layers | Mean = 0.47  Absolute Mean = 2.25 | Automatic |
| 7 | Sergiu Bilc et al  (2021)  (Romania) | Interleaving Automatic Segmentation and Expert Opinion for Retinal Conditions | 750 OCT B-Scan | GT+ WGD | 8 layers | SE  IPL-INL= −8.12$\pm$ 5.59  INL-OPL= −8.33 $\pm$7.68  OPL-ONL= −1.41 $\pm$3.51  MAE  IPL-INL= 12.67 $\pm$3.49  INL-OPL=7.88 $\pm$6.96  OPL-ONL=1.84 $\pm$3.28 | Automatic |
| 8 | Mansooreh Montazerin et al  (2021)  (Iran) | Livelayer: a semi-automatic software program for segmentation of layers and diabetic macular edema in optical coherence tomography images | Healthy and DME | Graph based semi-automatic method | 7 layers | Fluid objects (IRF and SRF) = 85.4% and the mean unsigned errors between the two algorithms was 1.53 for ILM and 1.33 for NFL–GCL | Semi-automatic |
| 9 | [Souvick Mukherjee](https://pubmed.ncbi.nlm.nih.gov/?term=Mukherjee%20S%5BAuthor%5D) et al.  (2022)  (USA) | Retinal layer segmentation in optical coherence tomography (OCT) using a 3D deep-convolutional regression network for patients with age-related macular degeneration | 358 eye volume AMD | 3D AggRegNet | 11 layers | MAE: 0.84$\pm$0.41  RMSE: 1.33$\pm$0.73 | Automatic |
| 10 | Xiang He et al  (2023)  (China) | Exploiting multi-granularity visual features for retinal layer segmentation in human eyes | 206 retinal images of healthy human eyes (named NR206 dataset) | A novel end-to-end retinal layer segmentation network based on ConvNeXt | 8 layers | Dice score = 91.3%, Mean IoU = 84.4%, ACC = 91.4 and mpa = 98.8 | Automatic |

*GT: Graph Theory, DP: Dynamic Programming, KR: kernel regression, WGD: weighted Geodesic Distance, CSCR: Central Serous Chorioretinopathy, SDOCT: Spectral-Domain Optical Coherence Tomography, MAE: Mean Absolute Error, GCL-IPL: Ganglion Cell Layer- Inner Plexiform Layer, OPL= Outer Plexiform Layer, RMSE; Root Mean Square Error, DME: Diabetic Macular Edema, AMD: Age-related Macular Degeneration,
